# Reliability of serological tests for COVID-19: comparison of three immunochromatography test kits for SARS-CoV-2 antibodies

**DOI:** 10.1016/j.heliyon.2020.e04929

**Published:** 2020-09-10

**Authors:** Hidetsugu Fujigaki, Masao Takemura, Michiko Osawa, Aki Sakurai, Kentaro Nakamoto, Koichi Seto, Takashi Fujita, Tadayoshi Hata, Hidehiko Akiyama, Yohei Doi, Kuniaki Saito

**Affiliations:** a Department of Disease Control and Prevention, Fujita Health University Graduate School of Health Sciences, 1-98 Dengakugakubo, Kutsukake-cho, Toyoake, Aichi, 470-1192, Japan; b Department of Joint Research Laboratory of Clinical Medicine, Fujita Health University Hospital, 1-98 Dengakugakubo, Kutsukake-cho, Toyoake, Aichi, 470-1192, Japan; c Department of Infectious Diseases, Fujita Health University School of Medicine, 1-98 Dengakugakubo, Kutsukake-cho, Toyoake, Aichi, 470-1192, Japan; d Center for Collaboration in Research and Innovation Research Promotion and Support Headquarters, Fujita Health University, 1-98 Dengakugakubo, Kutsukake-cho, Toyoake, Aichi, 470-1192, Japan; e Department of Molecular Laboratory Medicine, Fujita Health University Graduate School of Health Sciences, 1-98 Dengakugakubo, Kutsukake-cho, Toyoake, Aichi, 470-1192, Japan

**Keywords:** SARS-CoV-2, COVID-19, Antibody, Immunochromatography, Infectious disease, Virology, Immunology, Laboratory medicine, Diagnostics

## Abstract

**Background:**

Several immunochromatographic serological test kits have been developed to detect severe acute respiratory syndrome coronavirus 2 (SARS-CoV-2)-specific antibodies, but their relative performance and potential clinical utility is unclear.

**Methods:**

Three commercially available serological test kits were evaluated using 99 serum samples collected from 29 patients diagnosed with coronavirus disease 2019 (COVID-19) and 100 serum samples collected from 100 healthy volunteers in 2017 as negative controls.

**Results:**

The specificity of the IgM and IgG antibodies showed comparable results among the three immunochromatographic serological test kits. The specificity for IgM antibody was 98.0%, 98.0%, and 97.0%, and the specificity for IgG antibody was identical among the three kits (99.0%). The IgM antibody-positive rates of the three test kits for samples taken at the early stage of the disease (0–4 days after onset) were consistent with all three kits (18.2%); however, the IgM antibody-positive rates thereafter showed considerable differences among the kits, making it difficult to interpret the kinetics of IgM response against SARS-CoV-2. The IgG antibody-positive rates for samples taken after 13 days of onset were 100.0%, 97.6%, and 97.6%, respectively.

**Conclusion:**

There were large differences among the results of the three test kits. Only few cases showed positive results for IgM, suggesting that at least 2 of these kits used in this study were unsuitable for diagnosis of COVID-19. The IgG antibody was positive in almost all samples after 13 days of onset, suggesting that it may be useful for determining infections in the recent past.

## 1 Introduction

Coronavirus disease 2019 (COVID-19) has been spreading globally. COVID-19 is caused by severe acute respiratory syndrome coronavirus 2 (SARS-CoV-2) infection, which was confirmed around December 2019 in Wuhan, Hubei, China [1, 2]. Currently, real-time reverse transcription-polymerase chain reaction (RT-PCR) is employed to detect SARS-CoV-2 in a nasopharyngeal swab or sputum for the diagnosis of COVID-19 [3]. However, RT-PCR gives false-negative results in cases of a low viral titer and inadequate sample collection. Studies in China using RT-PCR tests reported that only 60%–70% of COVID-19 patients were positive in the early stages of infection [3, 4, 5, 6].

Detection of specific antibodies to a pathogen in the bloodstream is widely used to diagnose infectious diseases. An antibody test using a blood sample is relatively quick and straightforward, and because the risk of infection during the sample collection process is low, it is considered by some to be useful for the diagnosis of COVID-19 [7]. Generally, antigen-specific IgM antibodies increase in the early stage of the onset of a viral infection, which is then followed by an increased level of specific IgG antibodies. IgM antibodies are produced as the first antibody to fight the virus and are transiently raised. The subsequent production of IgG antibodies continues to rise for a long time and plays a vital role in immunity against the same virus. Therefore, the detection of specific IgM antibodies against SARS-CoV-2 may be used for diagnosis in the acute phase of COVID-19, whereas the detection of SARS-CoV-2-specific IgG antibodies may be used for determining a past infection or acquired immunity against SARS-CoV-2.

Immunochromatographic anti-SARS-CoV-2 antibody detection kits have been recently developed by multiple manufacturers [8]. However, differences in their properties and clinical usefulness are mostly unknown [9]. This study aimed to investigate the reliability of three different immunochromatographic anti-SARS-CoV-2 antibody detection kits using serum from COVID-19 patients.

## 2 Materials and methods

### 2.1 Detection of anti-SARS-CoV-2 antibodies

We evaluated three test kits for the detection of anti-SARS-CoV-2 IgM/IgG antibody in serum: 2019-nCoV IgG/IgM Rapid Test Cassette (Hangzhou AllTest Biotech Co., Ltd., China), COVID-19 IgM/IgG Duo (SD BIOSENSOR, Korea), and 2019-nCoV IgG/IgM Detection Kit (Vazyme Biotech Co., Ltd., China). All kits were based on colloidal gold-labeled immunochromatography. The colloidal gold-labeled immunochromatography test kit is a qualitative membrane-based immunoassay that detects antibodies in whole blood, serum, or plasma specimen. In the test component, anti-human IgG or IgM antibody is coated in each test line region. During testing, the specimen reacts with SARS-CoV-2 antigen-coated particles in the test cassette. The mixture then migrates upward on the membrane chromatographically via capillary action and reacts with the anti-human IgG or IgM antibodies in test line region. If the specimen contains IgG or IgM antibodies to SARS-CoV-2, a colored line appears in the test line region. According to the information provided by the manufacturers, Hangzhou AllTest and SD BIOSENSOR use nucleocapsid protein of SARS-CoV-2 as an antigen. The information about the antigen used in Vazyme Biotech kit is undisclosed. All tests were performed following the manufacturer's instructions for each kit, and the results were visually inspected within 15 min.

### 2.2 Study subjects

We utilized a series of residual serum samples left over after routine laboratory testing of 29 COVID-19 patients (mean age, 52.9 ± 21.9 years; 14 males and 15 females) who were admitted to Fujita Health University Hospital, Toyoake, Japan, from February 28 to April 15, 2020. All patients were confirmed as COVID-19 cases by RT-PCR assay of nasopharyngeal swab specimens at the time of or prior to admission. The date of onset was determined by review of electronic medical records as the day when they started experiencing symptoms of COVID-19.

One hundred serum samples from healthy human volunteers (mean age, 50.7 ± 10.0 years; 52 males and 48 females) were used as negative controls to evaluate the specificity of three test kits. All serum samples, aliquoted and stored at -80 °C, were thawed and evaluated at the same time for the analyses. This study was approved by the Ethics Committee for Clinical Research, Center for Research Promotion and Support, Fujita Health University (authorization number HM19-493 and HM17-341).

## 3 Results

### 3.1 Specificity of the three immunochromatographic kits

To test the specificity of three immunochromatographic kits, we utilized residual serum samples from 100 healthy donors as negative controls, which were collected between June 2017 and August 2017. As shown in Table 1, specificity for IgM antibody was 98.0% (Hangzhou AllTest), 98.0% (SD BIOSENSOR), and 97.0% (Vazyme Biotech). Two subjects as negative controls showed a positive result for IgM with all three kits. Vazyme Biotech showed lower specificity because one additional subject showed a positive result. These three subjects showed a positive result for only IgM.

**Table 1:** Specificity of anti-SARS-CoV-2 IgM and IgG antibodies of three immunochromatographic kits.

	IgM			IgG		
	No. of samples	No. of positive samples	Specificity (%)	No. of samples	No. of positive samples	Specificity (%)
Hangzhou AllTest	100	2	98.0	100	1	99.0
SD BIOSENSOR	100	2	98.0	100	1	99.0
Vazyme	100	3	97.0	100	1	99.0

Serum samples from healthy donors collected between June 2017 and August 2017 were tested as a negative control.

IgG antibody showed comparable results that revealed 99% specificity for all three kits (Table 1). One subject as negative control showed a positive result with all three kits for only IgG antibody.

### 3.2 Positive rate of anti-SARS-CoV-2 IgM antibody

Table 2 shows the results of anti-SARS-CoV-2 IgM antibody in patients’ serum according to the number of days after disease onset. The IgM antibody-positive rate of the all three kits in the early stage (0–4 days after onset) was 18.2%, showing consistent results among the kits. On the other hand, the IgM antibody-positive rate thereafter showed considerable differences among the kits (Table 2). For example, the IgM antibody-positive rates between 10 and 14 days after onset were 24.1%, 72.4%, and 17.2% for the Hangzhou AllTest, SD BIOSENSOR, and Vazyme kits, respectively. The SD BIOSENSOR kit showed a particularly high IgM antibody-positive rate throughout the entire period.

**Table 2:** Positive rate of anti-SARS-CoV-2 IgM antibody according to the different kits used.

IgM antibody							
Days after onset	No. of samples	Hangzhou AllTest		SD BIOSENSOR		Vazyme	
		No. of positive samples	Positive rate (%)	No. of positive samples	Positive rate (%)	No. of positive samples	Positive rate (%)
0–4	11	2	18.2	2	18.2	2	18.2
5–9	26	5	19.2	9	34.6	3	11.5
10–14	29	7	24.1	21	72.4	5	17.2
15–19	24	4	16.7	21	87.5	5	20.8
20–35	9	3	33.3	7	77.8	0	0.0

### 3.3 Positive rate of anti-SARS-CoV-2 IgG antibody

Table 3 shows the results of anti-SARS-CoV-2 IgG antibody in patients’ serum according to the number of days after onset. The results of IgG antibody from three kits tested in this study showed comparable results throughout the entire periods. The IgG antibody-positive rates of all three kits in the early stage (0–4 days after onset) were 27.3%, indicating that patients positive for IgG antibody were observed at the early stage of the onset. The IgG antibody-positive rates between 15 and 19 days after onset were 100% (Hangzhou AllTest), 95.8% (SD BIOSENSOR), and 95.8% (Vazyme). Thereafter all three kits showed 100% positivity for all samples collected between 20 and 35 days after the onset. Note that, since the IgM antibody-positive samples were also IgG antibody-positive, the positive rate of both IgM and IgG antibody was the same as the positive rate of the IgG antibody (Table 3).

**Table 3:** Positive rate of anti-SARS-CoV-2 IgG antibody according to the different kits used.

IgG antibody							
Days after onset	No. of samples	Hangzhou AllTest		SD BIOSENSOR		Vazyme	
		No. of positive samples	Positive rate (%)	No. of positive samples	Positive rate (%)	No. of positive samples	Positive rate (%)
0–4	11	3	27.3	3	27.3	3	27.3
5–9	26	11	42.3	11	42.3	10	38.5
10–14	29	24	82.8	23	79.3	22	75.9
15–19	24	24	100.0	23	95.8	23	95.8
20–35	9	9	100.0	9	100.0	9	100.0
After 13	42	42	100.0	41	97.6	41	97.6
Total	99	71	71.7	69	69.7	67	67.7

The positive rate of both IgM and IgG antibody was the same as the positive rate of the IgG antibody.

### 3.4 Kinetics of anti-SARS-CoV-2 IgM and IgG antibody

**Figure 1:**
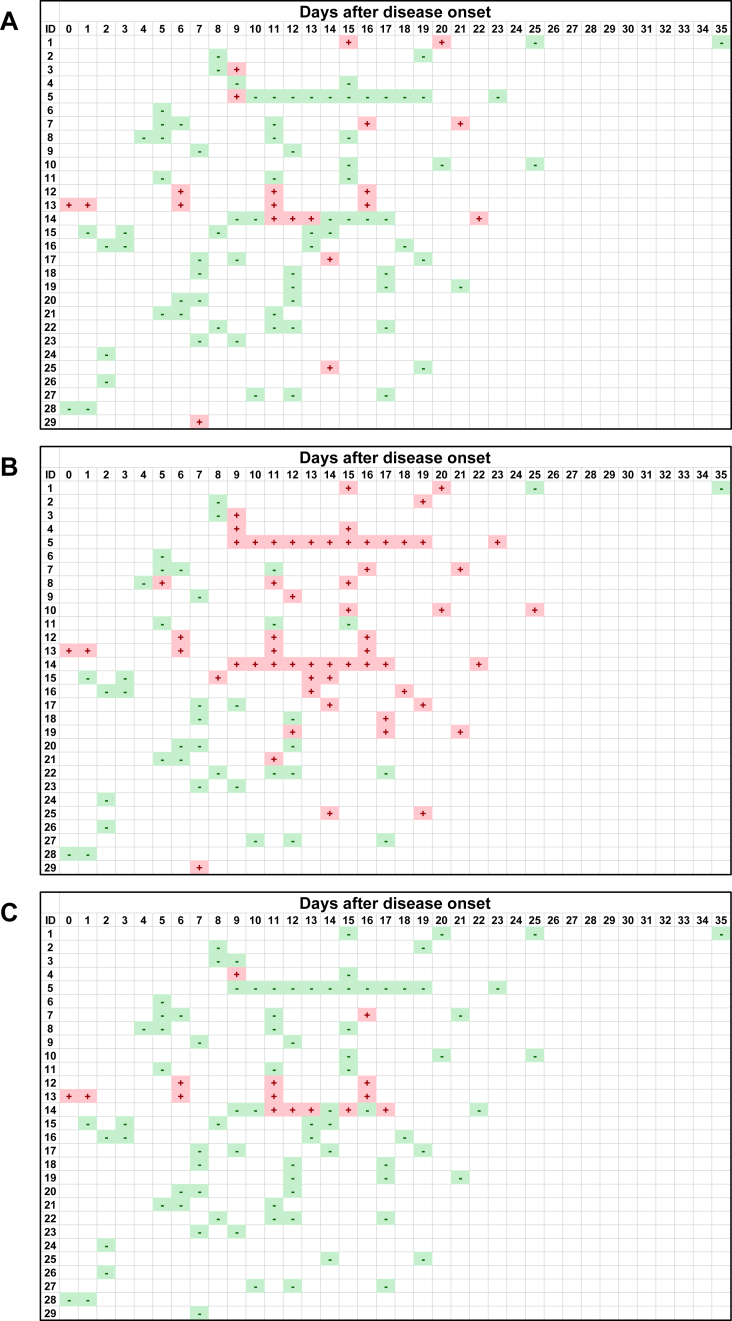
**Kinetics of anti-SARS-CoV-2 IgM antibody of serum samples from 29 COVID-19 patients**. The positive or negative result of the antibody in each serum sample is expressed as + or −, respectively. A, Hangzhou AllTest; B, SD BIOSENSOR; C, Vazyme.

**Figure 2:**
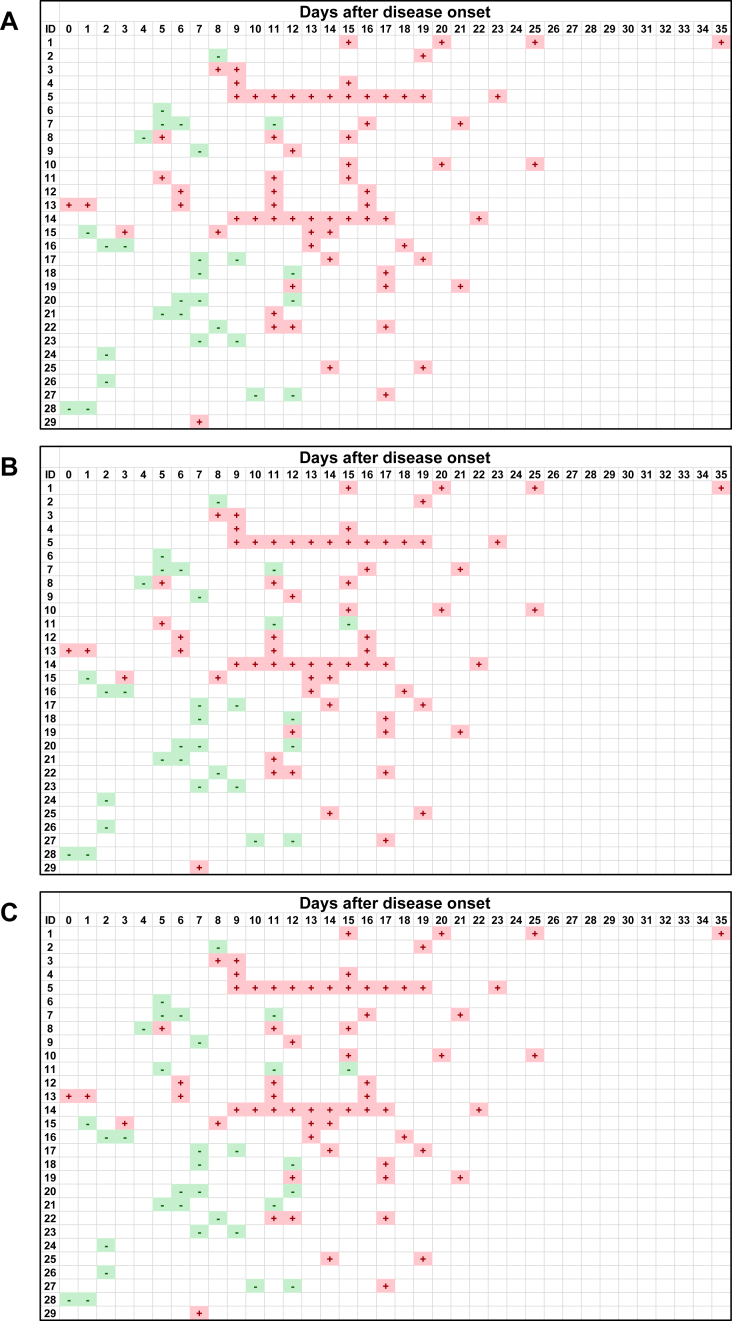
**Kinetics of anti-SARS-CoV-2 IgG antibody of serum samples from 29 COVID-19 patients**. The positive or negative result of the antibody in each serum sample is expressed as + or −, respectively. A, Hangzhou AllTest; B, SD BIOSENSOR; C, Vazyme.

Figures 1 and 2 show the kinetic results of anti-SARS-CoV-2 IgM and IgG antibody of the 29 patients in our cohort. While most samples collected within 7 days after the onset showed IgM negativity in all three kits, the IgM positivity increased between 8 and 10 days after the onset in all three test kits. It appears that the IgM positivity became negative at 18 and 22 days after the onset in Hangzhou AllTest and Vazyme, respectively, while the IgM positivity in SD BIOSENSOR continued to be positive until at least 25 days after the onset. The different results among the three test kits and the variation of intervals and number of samples from each patient made it difficult to interpret the kinetic pattern of IgM. Furthermore, there was a dissociation of the results in patient 14, where the SD BIOSENSOR gave consistently positive results, whereas the Hangzhou AllTest and Vazyme kits gave intermittently positive and negative results. No samples showed IgM positivity prior to IgG positivity, indicating that IgM seroconversion does not precede IgG seroconversion.

Conversely, tests for the IgG antibody often turned positive about 10 days after the onset, and most of the samples after 13 days of the onset became positive with all kits. However, there was a dissociation of the results in patient 11, where the Hangzhou AllTest kit gave positive results in all samples, whereas the SD BIOSENSOR and Vazyme kits gave negative results.

## 4 Discussion

The serological detection of antibodies is widely used for the diagnosis of viral infections. Since immunochromatography methods to detect antibodies are quick to perform, easy to use, and do not require additional equipment, detection of anti-SARS-CoV-2 antibody using immunochromatography kits may be helpful for diagnosing COVID-19.

We compared three immunochromatography kits for detecting anti-SARS-CoV-2 antibodies using serum samples from COVID-19 patients. There was a considerable difference among the kit results, especially for IgM (Table 2 and Figure 1). SD BIOSENSOR showed highest positivity rate for IgM among the three test kits, although the specificity was comparable among the kits (Tables 1 and 2). This indicates that, at least among the tested kits in this study, SD BIOSENSOR is most reliable kit for detecting anti-SARS-CoV-2 IgM antibody. Furthermore, there was a dissociation of the results in patient 14, where the SD BIOSENSOR gave consistently positive results, whereas the Hangzhou AllTest and Vazyme kits gave intermittently positive and negative results (Figure 1). We speculate that this is caused by the difference of the limit of detection among the three kits. SD BIOSENSOR seems to have lowest limit of detection among the three kits, it may contribute to the highest sensitivity of IgM among the three kits. Further studies, such as quantification of the antibodies, are needed to clarify this issue. Anyway, it is thus clear that kit selection is crucial and should be based on the clinical purpose for the test. Additionally, since only a few samples were positive for the IgM antibody in the early stage of the disease, it appears that the kits used in this study are unsuitable for diagnosing the acute phase of COVID-19.

However, this study has some limitations to interpret the results of IgM antibody. The intervals of sampling and number of samples from each patient vary among patients. Also, there are several anti-SARS-CoV-2 antibody test kits that have been made available since the past spring; comparison study of the tested kits with these new kits using a large number of sample which are taken consistently after the onset is necessary.

A comparison of the IgG antibody results between the three kits did not reveal any large differences. Additionally, almost all serum samples collected after 13 days of onset were positive (Figure 2). This suggests that the IgG antibody test using these kits can be helpful in diagnosing COVID-19 after a certain period from disease onset. However, the results of patient 11 were positive for one kit and negative for the other two kits, suggesting the presence of false-positive and false-negative results (Figure 2). Since the specificity for IgG antibody among three kits were identical (Table 1), we cannot conclude whether Hangzhou AllTest showed false-positive and the other two showed true negative, or vice versa. Consistent with this study, the presence of false-positive results of immunochromatography anti-SARS-CoV-2 antibody test kits has been reported [10, 11]. It has been reported that serum from patients with rheumatoid arthritis and Sjogren's syndrome showed a false-positive result for anti-SARS-CoV-2 IgM antibody using immunochromatography kits [11, 12]. However, since the cause of a false-positive result for anti-SARS-CoV-2 IgG antibody is unknown, further investigation is necessary, such as determining the presence of cross-reactions with other coronaviruses. Furthermore, it is unclear how long anti-SARS-CoV-2 IgG antibody levels continue to rise in serum after infection, and further research is warranted.

Currently, epidemiological studies in several countries use immunochromatography kits to detect antibodies [13]. We suggest that when using immunochromatography kits for COVID-19 diagnosis, particular attention must be paid to whether these kits detect neutralizing antibodies against SARS-CoV-2. SARS-CoV-2 contains at least four structural proteins (spike [S] protein, envelope protein, membrane protein, and nucleocapsid [N] protein) [14, 15]. To the best of our knowledge, Hangzhou AllTest and SD BIOSENSOR use N protein as an antigen. Since receptor binding domain (RBD) in S protein appears to be involved in adhesion between virus and host cells during infection, it is considered that an antibody against RBD acts as a neutralizing antibody [16, 17, 18]. The detection of antibodies by immunochromatography kits may be useful for determining a previous virus infection, but it is unclear whether the results could indicate people who have gained acquired immunity. Most recently, it has been reported that several companies developed test kits to quantify neutralizing antibodies against SARS-CoV-2 RBD [19]. This study indicated that the antibodies detected by these methods in the convalescent plasma of COVID-19 patients correlated with neutralization activity. Besides, quantitative assay of anti-SARS-CoV-2 antibodies, such as enzyme-linked immunosorbent assay (ELISA) and chemiluminescent enzyme immunoassay (CLEIA), would give a better understanding of humoral immune response to COVID-19 infection over time.

## Declarations

### Author contribution statement

Hidetsugu Fujigaki: Conceived and designed the experiments; Performed the experiments; Analyzed and interpreted the data; Wrote the paper.

Masao Takemura, Kentaro Nakamoto: Conceived and designed the experiments; Performed the experiments.

Michiko Osawa: Performed the experiments; Contributed reagents, materials, analysis tools or data.

Aki Sakurai: Conceived and designed the experiments; Analyzed and interpreted the data; Contributed reagents, materials, analysis tools or data; Wrote the paper.

Koichi Seto, Takashi Fujita, Tadayoshi Hata, Hidehiko Akiyama: Analyzed and interpreted the data; Contributed reagents, materials, analysis tools or data.

Yohei Doi: Conceived and designed the experiments; Analyzed and interpreted the data; Contributed reagents, materials, analysis tools or data; Wrote the paper.

Kuniaki Saito: Conceived and designed the experiments; Analyzed and interpreted the data; Contributed reagents, materials, analysis tools or data.

### Funding statement

This research did not receive any specific grant from funding agencies in the public, commercial, or not-for-profit sectors.

### Competing interest statement

The authors declare the following conflict of interests: Hidetsugu Fujigaki received immunochromatographic anti-SARS-CoV-2 antibody detection kits from Nichirei Biosciences Inc. and Shionogi & Co., Ltd.

### Additional information

No additional information is available for this paper.
